# A case report of *Salmonella enterica* serovar Corvallis from environmental isolates from Cambodia and clinical isolates in the UK

**DOI:** 10.1099/acmi.0.000315

**Published:** 2022-01-12

**Authors:** Carla L. Schwan, Timothy J. Dallman, Peter W. Cook, Jessie Vipham

**Affiliations:** ^1^​ Department of Nutritional Sciences, University of Georgia, 300 Carlton St., Athens, GA 30602, USA; ^2^​ National Infection Service, Public Health England, London, UK; ^3^​ Centers for Disease Control and Prevention, Atlanta, Georgia, USA; ^4^​ Department of Animal Sciences and Industry, Food Science Institute, Kansas State University, Manhattan, KS, USA

**Keywords:** non-typhoidal *Salmonella*, Cambodia, informal markets, clinical isolates, *Salmonella enterica *serovar Corvallis, traveller

## Abstract

*
Salmonella enterica* subspecies *enterica* serovar Corvallis (*S*. Corvallis) has been identified as a human pathogen and as a food contaminant. Diarrhoeal disease is a common diagnosis in tourists visiting Southeast Asia, often with unknown aetiology. However, numerous public health institutes have identified *Salmonella* as a common causative agent when consuming contaminated food and water. Genomic data from environmental isolates from a Cambodian informal market were uploaded to the National Center for Biotechnology Information (NCBI) platform, allowing the novel sequences to be compared to global whole-genome sequence archives. The comparison revealed that two human clinical isolates from England and four of the environmental isolates were closely related, with an average single nucleotide polymorphism (SNP) difference of 1 (0–3 SNPs). A maximum-likelihood tree based on core SNPs was generated comparing the 4 isolates recovered from a Cambodian informal market with 239 isolates of *S*. Corvallis received from routine surveillance of human salmonellosis in England and confirmed the close relationship. In addition, the environmental isolates clustered into a broader phylogenetic group within the *S*. Corvallis population containing 68 additional human isolates, of which 42 were from patients who reported recent international travel, almost exclusively to Southeast Asia. The environmental isolates of *S*. Corvallis isolated from an informal market in Cambodia are concerning for public health due to their genetic similarity to isolates (e.g. clinical isolates from the UK) with known human virulence and pathogenicity. This study emphasizes the benefits of global and public data sharing of pathogen genomes.

## Introduction

*Salmonella enterica* (henceforth referred to as *Salmonella *) is the primary causative agent of diarrhoeal diseases worldwide, with the most severe effects seen in low- and middle-income countries (LMICs; e.g. Southeast Asian countries) [1]. *Salmonella* are ubiquitous bacteria, with over 2500 different serovars reported to date [2]. A pathogenic serovar of interest, *Salmonella enterica* serovar Corvallis (*S*. Corvallis), has been reported worldwide in humans and food products [3–6], and is among the top 10 most frequently isolated serovars from human patients in Southeast Asia [3].

International travellers who visit Southeast Asian countries (e.g. Cambodia, Thailand and Vietnam) [7] can potentially develop diarrhoeal diseases, such as salmonellosis, by coming into contact with contaminated food and water [8, 9]. Furthermore, *S*. Corvallis has been identified as a common causative agent for travel-related salmonellosis (i.e. travel to Southeast Asian countries) by numerous public health institutes [5, 10–12].

Characterization of *Salmonella* serovars can be accomplished by whole-genome sequencing (WGS), which is a powerful tool to aid in the identification of bacterial isolates similarities [13] and source attribution pathways (i.e. methods used to attribute human cases of foodborne diseases to a specific source, such as microbial subtyping, outbreak summary data, epidemiological studies) [14]. Here, we report the genetic relatedness of *S*. Corvallis from environmental samples from Cambodia and from clinical isolates in the UK through WGS.

## Case report

Public Health England (PHE) adopted routine WGS for the identification and characterization of referred *Salmonella* isolates in April 2014 [15, 16] and genomes were deposited in the National Center for Biotechnology Information (NCBI) BioProject PRJNA248792. As of December 2020, 239 isolates have been typed as *S*. Corvallis, of which 115 were from isolates that reported international travel prior to onset of symptoms.

In January 2019, a group of researchers from Kansas State University travelled to Cambodia to investigate the prevalence of *Salmonella* on environmental surfaces in informal markets [17]. *Salmonella* was isolated from environmental samples and shipped to the USA for WGS analysis and characterization [18]. In November of 2019, the Center for Food Safety and Applied Nutrition (CFSAN) at the US Food and Drug Administration (FDA) conducted WGS analysis and uploaded those sequences to the NCBI platform.

## Methodology

This paper involves the reanalysis of previously published and freely available genome sequences [18]. An SNP analysis was conducted on the NCBI Pathogen Detection platform (https://www.ncbi.nlm.nih.gov/pathogens/) to identify isolate similarities between the Cambodia environmental isolate genomes and all other genomes available in the library. The NCBI Pathogen Detection platform was used to evaluate the whole-genome sequences in the context of phylogenetic trees using isolate sequences that have been uploaded to the NCBI’s library [19]. The NCBI’s Pathogen Detection platform builds phylogenetic trees by first comparing the k-mer distance of whole-genome sequences and then building SNP matrices for comparing differences in SNPs among different closely related isolates [https://www.ncbi.nlm.nih.gov/pathogens/pathogens_help/#data-processing]. The SNPs represent genetic variation and are commonly used to compare the genetic relatedness and evolutionary origin in a bacterial population [20]. The SNP analysis was retrieved from the NCBI Pathogen Detection using the Isolates SNP Tree Viewer [21, 22]. If an isolate belongs to an SNP cluster (i.e. a group of isolates in which genome assemblies are clustered) it has a link to the SNP Tree Viewer. The SNP Tree Viewer is then built using the maximum compatibility method [19] and displays relationships among the isolates based upon the number of SNPs they contain relative to each other. K-mer comparisons are used to generate clusters of bacterial isolates with no more than 50 SNPs. The SNPs are called by the Pathogen Detection Pipeline by making pairwise comparisons to all genomes in a given cluster. The cluster-specific reference is used to identify all positions that have at least one high-quality SNP located in another isolate from the cluster. SNPs are considered to be high quality if they are not identified in repeat regions, from recombination events, associated with phages, or artefacts of the assembly process. The identified clusters are reconstructed into phylogenetic trees using the identified set of high-quality SNPs and the single reference from the cluster to compute the maximum compatibility algorithm described by Cherry 2018 [19].

A second phylogenetic analysis was performed using high-quality reads mapped to the reference *Salmonella* Corvallis isolate 12–01738, (GenBank accession CP027677), using the default options in Burrows–Wheeler Aligner – Maximum Exact Matching [BWA MEM (v0.7.2)] [23]. The sequence alignment map output from BWA was sorted and indexed to produce a binary alignment map (BAM) using Samtools (v1.1) [24]. Genome Analysis Toolkit (GATK v2.6.5) was then used to create a variant call format (VCF) file from each of the sorted BAMs, which were further parsed to extract only SNP positions of high quality [mapping quality (MQ) >30, depth (DP) >10, variant ratio >0.9]. SNP alignments were created tolerating positions where >80 % of isolates had a base call with regions of recombination masked using default options in Gubbins v2.0.0 [25]. Maximum-likelihood phylogenies were computed using IQ-TREE v2.0.4 [26] with the best-fit model automatically selected and near zero branches collapsed into polytomies.

## Findings

It was discovered using the SNP analysis that several of the environmental isolates (*n*=4) identified as *S*. Corvallis were closely related to two clinical isolates submitted by PHE in 2016 and 2019. The 2016 isolate was recovered from a 20-year-old native British woman who had travelled to Vietnam prior to the onset of symptoms. The 2019 isolate of *S*. Corvallis was from a 60-year-old native British man with no report of travel prior to onset of symptoms. No specific consent was required from the patients whose data were used in this analysis because PHE has authority to handle patient data for public health monitoring and infection control under section 251 of the UK National Health Service Act of 2006.

An SNP difference of two nucleotides between the clinical isolate (PDT000475677.1) and four environmental isolates (PDT000630248.1, PDT000630194.1, PDT000630209.1 and PDT000630173.1) is shown in Table 1. Further, a SNP difference of two and zero nucleotides was identified between the clinical isolate (PDT000522124.1) and the same environmental isolates (Table 1).

**Table 1.** *
Salmonella enterica* isolates from Cambodia (environmental), the UK (clinical) and the USA (clinical) within the same SNP cluster (PDS000056420.14)

Serovar*	Isolate	Create date†	Location	Isolation type	Min–same‡	Min–diff§	NCBI accession no.
**Year: 2019**							
||Corvallis	PDT000457118.1	31 Jan 2019	UK	Clinical	8	15	SAMN10848942
||Corvallis	PDT000469598.1	27 Feb 2019	UK	Clinical	4	16	SAMN11025553
||Corvallis	PDT000470700.1	28 Feb 2019	UK	Clinical	7	14	SAMN11031785
||Corvallis	PDT000471288.1	2 Mar 2019	UK	Clinical	3	10	SAMN11042238
Corvallis	PDT000475677.1	9 Mar 2019	UK	Clinical	2	2	SAMN11093783
||Corvallis	PDT000475844.1	10 Mar 2019	UK	Clinical	3	8	SAMN11095741
||Corvallis	PDT000477511.1	15 Mar 2019	UK	Clinical	5	16	SAMN11128906
||Corvallis	PDT000477513.1	15 Mar 2019	UK	Clinical	5	12	SAMN11128913
Corvallis	PDT000522124.1	12 June 2019	UK	Clinical	2	0	SAMN12039715
||Corvallis	PDT000470764.1	1 Mar 2019	UK	Clinical	6	17	SAMN11038684
Corvallis	PDT000630173.1	19 Nov 2019	Cambodia	Environmental	0	0	SAMN13321508
Corvallis	PDT000630194.1	19 Nov 2019	Cambodia	Environmental	0	0	SAMN13321598
Corvallis	PDT000630209.1	19 Nov 2019	Cambodia	Environmental	0	0	SAMN13322169
Corvallis	PDT000630248.1	19 Nov 2019	Cambodia	Environmental	1	1	SAMN13322423
||Corvallis	PDT000639610.1	4 Dec 2019	UK	Clinical	8	17	SAMN13474428
**Year: 2018**							
||Corvallis	PDT000317841.1	22 May 2018	UK	Clinical	4	11	SAMN09233616
||Corvallis	PDT000319348.1	25 May 2018	UK	Clinical	4	10	SAMN09257893
||Corvallis	PDT000323021.1	3 June 2018	UK	Clinical	6	13	SAMN09298374
||Corvallis	PDT000327612.1	10 June 2018	UK	Clinical	2	11	SAMN09388926
||Corvallis	PDT000332213.1	19 June 2018	UK	Clinical	7	14	SAMN09444533
||Corvallis	PDT000336426.1	26 June 2018	UK	Clinical	5	15	SAMN09484468
||Corvallis	PDT000338111.1	28 June 2018	UK	Clinical	3	11	SAMN09504423
||Corvallis	PDT000342475.1	6 July 2018	UK	Clinical	2	11	SAMN09624182
||Corvallis	PDT000377617.1	15 Sept 2018	UK	Clinical	4	16	SAMN10067798
||Corvallis	PDT000379076.1	17 Sept 2018	UK	Clinical	4	13	SAMN10076048
||Corvallis	PDT000312919.2	10 May 2018	UK	Clinical	6	17	SAMN09100973
||Corvallis	PDT000340695.1	3 July 2018	UK	Clinical	9	16	SAMN09534667
**Year: 2017**							
||na	PDT000230176.2	1 Aug 2017	USA	Clinical	7	14	SAMN07277150
||Corvallis	PDT000214300.2	31 May 2017	UK	Clinical	7	15	SAMN07180127
||na	PDT000215685.2	5 June 2017	USA	Clinical	4	11	SAMN07173395
**Year: 2016**							
||Corvallis	PDT000129606.2	10 May 2016	USA	Clinical	3	10	SAMN04913844
||Chailey	PDT000103370.2	26 Jan 2016	UK	Clinical	0	7	SAMN04437636
**Year: 2015**							
||Corvallis	PDT000639610.1	4 Dec 2019	UK	Clinical	8	17	SAMN13474428
||Corvallis	PDT000042987.4	10 Feb 2015	UK	Clinical	9	10	SAMN03168761
||Corvallis	PDT000043024.4	10 Feb 2015	UK	Clinical	4	11	SAMN03168799
||Chailey	PDT000058820.2	12 Apr 2015	UK	Clinical	0	7	SAMN03479345
||Chailey	PDT000059500.2	13 Apr 2015	UK	Clinical	0	7	SAMN03479962
||Corvallis	PDT000053912.2	10 Apr 2015	UK	Clinical	6	17	SAMN03468587
**Year: 2014**							
||Corvallis	PDT000040841.2	17 Oct 2014	USA	Clinical	6	13	SAMN03098691

*Phenotypic characterization of isolates.

†Corresponds to the date the genomes were uploaded to the NCBI.

‡Min–same: minimum SNP distance from this isolate to another isolate of the same isolation type.

§Min–diff: minimum SNP distance from this isolate to another isolate of a different isolation type.

||Supplementary information on clinical patients not available.

Although supplementary information on additional clinical patients was not available, the SNP analysis also revealed 4 clinical isolates in the USA and an additional 29 clinical isolates in the UK that shared similar genetic profiles (SNP difference ranging from 0 to 14) with the environmental isolates from Cambodia. These clinical isolates were reported between 2014–2019, as shown in Table 1. Three closely related isolates from England were reported as *S*. Chailey defined phenotypically, although genotypically they are consistent with them being in the *S*. Corvallis group.

**Fig. 1.**
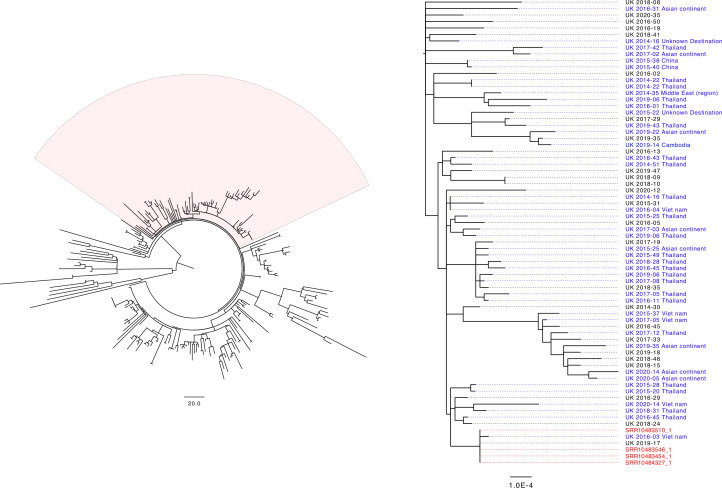
**(a**) Maximum-likelihood tree based on 3698 core SNPs generated with IQTREE2 of 239 *S*. Corvallis genomes received by PHE since April 2014. Highlighted region represents monophyletic cluster associated with travel to Southeast Asia. The scale bar represents the number of nucleotide substitutions. (b) Maximum-likelihood tree based on 554 core SNPs generated with IQTREE2 of 72 *S*. Corvallis genomes from the Southeast Asian clade; the highlighted region represents the monophyletic cluster that contains the 4 isolates recovered from a Cambodian informal market (red). UK isolates annotated with year and week number and isolates with reported travel before onset of symptoms are highlighted in blue. The scale bar represents nucleotide substitutions per site.

Genomes from *S*. Corvallis isolates from informal markets in Cambodia were also compared to the 239 isolates of *S*. Corvallis received from routine surveillance in England using the PHE SnapperDB pipeline [27]. The Cambodian isolates clustered with the two human clinical isolates described above. In addition, the Cambodian isolates clustered into a broader phylogenetic group within the *S*. Corvallis population containing 68 additional human isolates, of which 42 were from patient who reported recent international travel, almost exclusively to Southeast Asia (Fig. 1, Table S1, available in the online version of this article).

## Discussion

Sequence similarity of pathogen genomes can infer the relatedness between isolates as the fewer genetic differences identified between pairs of isolates, the less time since divergence from a common ancestor. As such, isolates with very similar genomes have an increased likelihood that they are transmitted via the same vehicle and/or from the same source population. In this report, an average SNP difference of 1 (0–3 SNPs) between the 2016 and 2019 clinical isolates and the environmental isolates was observed, indicating a high level of genetic similarity. Interestingly, the additional 33 clinical isolates from 2 countries (i.e. the USA and UK) were also considered to be genetically related and ranged from 0 to 10 SNPs different relative to the environmental isolates from Cambodia. These results reveal that this pathogenic strain of *S*. Corvallis was recurrently associated with human disease since 2014.

Human infections caused by *S*. Corvallis have mostly been reported in Asian countries with relatively few infections reported outside this region (e.g. PR China, Thailand, Vietnam and Malaysia [28–31]. In fact, *S*. Corvallis is among the top 10 most frequently isolated serotypes from human patients in Southeast Asia [3]. For example, a case of travel-related bacteraemia caused by *S*. Corvallis was reported in an immunocompetent adult from Japan who had travelled to Cambodia and Vietnam prior the beginning of symptoms [5]. These reports indicate that this serovar may be endemic in geographical areas of Asia.

*S*. Corvallis has been isolated from food products and environments (food contact and non-food contact surfaces) in Southeast Asia, the USA, Brazil, North Africa and Europe [3, 17, 32]. Reports of highly antibiotic-resistant isolates of *S*. Corvallis have raised concerns within public health authorities around the world [33–35]. Further, multidrug-resistant *S*. Corvallis was previously recovered from patients with a history of travel to Vietnam and Thailand [12].

In Cambodia, studies revealed the presence of *S*. Corvallis in poultry [4], pork [6] and environmental [17] samples from retail and informal markets. The informal markets are characterized by open-air environments that lack basic food safety infrastructure, hygiene, sanitation or oversight [36]. Informal markets are often a tourist attraction for international travellers, who frequently purchase and consume food products at these locations. Reports indicate that the consumption of contaminated food and water has resulted in traveller’s diarrhoea cases worldwide [8, 37]. In fact, the risk of *Salmonella* infection in travellers returning to the USA is highest for travellers returning from Africa, Latin America and Asia [8].

The environmental isolates presented in this report (isolated from surfaces in informal markets in Cambodia) share high genetic similarity to the human clinical strain in the UK. *S*. Corvallis from clinical isolates have been reported in the USA (in 2014, 2016 and 2017) and in the UK (in 2014, 2015, 2016, 2017, 2018 and 2019) for many consecutive years, indicating that this lineage poses a recurrent threat of infection in humans. Although *S*. Corvallis has caused bacteraemia and diarrhoeal disease [5, 12], few studies have investigated its source of contamination and routes of transmission. Therefore, public health will benefit if future studies focus on the contamination and transmission routes of this pathogen.

This report indicates that these isolates of *S*. Corvallis isolated from environmental surfaces in informal markets in Cambodia are concerning for public health because of their genetic similarity to isolates that have caused human disease. Since the majority of the Cambodian population acquires their food products from these informal markets, it is important to focus on strategies to control and prevent the contamination of *S*. Corvallis (along with other pathogenic serovars of *Salmonella *) in these locations. Future work should focus on source attribution and persistence studies identifying common sources of contamination and determining major transmission routes.

## Supplementary Data

Supplementary material 1
