# Supplementary material for: A case report of Salmonella enterica serovar Corvallis from environmental isolates from Cambodia and clinical isolates in the UK
Source: Access Microbiol. 2022 Jan 12;4(1):000315. doi: 10.1099/acmi.0.000315 (PMC8895601; doi:10.1099/acmi.0.000315)
Supplement: Supplementary material 1 [file acmi-4-0315-s001.pdf]

Supplementary Table 1. Accession number, tree label, and travel history corresponding to the isolates used to create Figure 1.

Accession numbers	Tree label	Travel history
SRR7348920	UK - Background	Vietnam
SRR8508565	UK - Background	Vietnam
SRR1965115	UK - Background	Unknown Destination
SRR10285461	UK - Background	Thailand
SRR15361945	UK - Background	Thailand
SRR15371366	UK - Background	Thailand
SRR1966703	UK - Background	Thailand
SRR1966952	UK - Background	Thailand
SRR1968335	UK - Background	Thailand
SRR3049005	UK - Background	Thailand
SRR3322120	UK - Background	Thailand
SRR5632821	UK - Background	Thailand
SRR5632925	UK - Background	Thailand
SRR7292859	UK - Background	Thailand
SRR7456872	UK - Background	Thailand
SRR7458001	UK - Background	Thailand
SRR7998245	UK - Background	Thailand
SRR8149161	UK - Background	Thailand
SRR8485109	UK - Background	Thailand
SRR3321846	UK - Background	Sudan
SRR7471134	UK - Background	Sri lanka
SRR8149425	UK - Background	Sri lanka
SRR8325573	UK - Background	Sri lanka
SRR8397144	UK - Background	Sri lanka
SRR8490758	UK - Background	Sri lanka
SRR3322964	UK - Background	Spain
SRR1963267	UK - Background	Singapore
SRR8524727	UK - Background	Singapore
SRR3323004	UK - Background	Philippines
SRR7310597	UK - Background	Peru
SRR7528157	UK - Background	Pakistan
SRR11410454	UK - Background	Morocco
SRR1967851	UK - Background	Morocco
SRR7090650	UK - Background	Morocco
SRR7159871	UK - Background	Morocco
SRR7426917	UK - Background	Morocco

SRR8701061	UK - Background	Morocco
SRR8131510	UK - Background	Mexico
SRR10294385	UK - Background	Malta
SRR1958086	UK - Background	Malaysia
SRR7223257	UK - Background	Malaysia
SRR7359048	UK - Background	Malaysia
SRR7469113	UK - Background	Malaysia
SRR8691504	UK - Background	Malaysia
SRR10247583	UK - Background	Italy
SRR1967381	UK - Background	Indonesia
SRR3322440	UK - Background	Indonesia
SRR3322647	UK - Background	Indonesia
SRR7850645	UK - Background	Indonesia
SRR7187975	UK - Background	India
SRR7286868	UK - Background	Hong kong
SRR1966528	UK - Background	Greece
SRR1958019	UK - Background	Dominican republic
SRR3321899	UK - Background	Dominican republic
SRR7192176	UK - Background	Dominican republic
SRR7416343	UK - Background	Dominican republic
SRR9054506	UK - Background	Cuba
SRR9287438	UK - Background	Cuba
SRR3321875	UK - Background	Colombia
SRR7495767	UK - Background	Colombia
SRR7997148	UK - Background	Bulgaria
SRR7450700	UK - Background	Australia
SRR1965162	UK - Background	Asian continent
SRR1968642	UK - Background	Asian continent
SRR1969856	UK - Background	Asian continent
SRR3322058	UK - Background	Asian continent
SRR7187981	UK - Background	Asian continent
SRR7188004	UK - Background	Asian continent
SRR7292909	UK - Background	Asian continent
SRR7890230	UK - Background	Asian continent
SRR8098362	UK - Background	Asian continent
SRR8427350	UK - Background	Asian continent
SRR9153233	UK - Background	Asian continent
SRR10018142	UK - Background	Not recorded
SRR10096385	UK - Background	Not recorded
SRR10285463	UK - Background	Not recorded

SRR10302536	UK - Background	Not recorded
SRR10542572	UK - Background	Not recorded
SRR10723216	UK - Background	Not recorded
SRR10992269	UK - Background	Not recorded
SRR11041614	UK - Background	Not recorded
SRR11142799	UK - Background	Not recorded
SRR11362263	UK - Background	Not recorded
SRR12770288	UK - Background	Not recorded
SRR1958452	UK - Background	Not recorded
SRR1963274	UK - Background	Not recorded
SRR1963278	UK - Background	Not recorded
SRR1965198	UK - Background	Not recorded
SRR1965504	UK - Background	Not recorded
SRR1967891	UK - Background	Not recorded
SRR1968128	UK - Background	Not recorded
SRR1968538	UK - Background	Not recorded
SRR1968623	UK - Background	Not recorded
SRR1969034	UK - Background	Not recorded
SRR1969580	UK - Background	Not recorded
SRR3322388	UK - Background	Not recorded
SRR3322671	UK - Background	Not recorded
SRR5583854	UK - Background	Not recorded
SRR5584688	UK - Background	Not recorded
SRR5631651	UK - Background	Not recorded
SRR5633371	UK - Background	Not recorded
SRR6324225	UK - Background	Not recorded
SRR6868294	UK - Background	Not recorded
SRR7122397	UK - Background	Not recorded
SRR7140635	UK - Background	Not recorded
SRR7157776	UK - Background	Not recorded
SRR7167592	UK - Background	Not recorded
SRR7180173	UK - Background	Not recorded
SRR7188021	UK - Background	Not recorded
SRR7204605	UK - Background	Not recorded
SRR7251539	UK - Background	Not recorded
SRR7284353	UK - Background	Not recorded
SRR7284365	UK - Background	Not recorded
SRR7285606	UK - Background	Not recorded
SRR7348944	UK - Background	Not recorded
SRR7349235	UK - Background	Not recorded

SRR7369163	UK - Background	Not recorded
SRR7401756	UK - Background	Not recorded
SRR7402036	UK - Background	Not recorded
SRR7402055	UK - Background	Not recorded
SRR7402402	UK - Background	Not recorded
SRR7410339	UK - Background	Not recorded
SRR7415100	UK - Background	Not recorded
SRR7416285	UK - Background	Not recorded
SRR7416346	UK - Background	Not recorded
SRR7426524	UK - Background	Not recorded
SRR7439298	UK - Background	Not recorded
SRR7450791	UK - Background	Not recorded
SRR7450809	UK - Background	Not recorded
SRR7450856	UK - Background	Not recorded
SRR7451142	UK - Background	Not recorded
SRR7451151	UK - Background	Not recorded
SRR7458190	UK - Background	Not recorded
SRR7458199	UK - Background	Not recorded
SRR7458664	UK - Background	Not recorded
SRR7458809	UK - Background	Not recorded
SRR7474663	UK - Background	Not recorded
SRR7474989	UK - Background	Not recorded
SRR7522887	UK - Background	Not recorded
SRR7523118	UK - Background	Not recorded
SRR7523649	UK - Background	Not recorded
SRR7538778	UK - Background	Not recorded
SRR7828408	UK - Background	Not recorded
SRR7828508	UK - Background	Not recorded
SRR7841369	UK - Background	Not recorded
SRR7850548	UK - Background	Not recorded
SRR7885254	UK - Background	Not recorded
SRR7890212	UK - Background	Not recorded
SRR7892134	UK - Background	Not recorded
SRR7903060	UK - Background	Not recorded
SRR8084262	UK - Background	Not recorded
SRR8087185	UK - Background	Not recorded
SRR8098330	UK - Background	Not recorded
SRR8106807	UK - Background	Not recorded
SRR8116992	UK - Background	Not recorded
SRR8382444	UK - Background	Not recorded

SRR8441122	UK - Background	Not recorded
SRR8485189	UK - Background	Not recorded
SRR8528765	UK - Background	Not recorded
SRR8543264	UK - Background	Not recorded
SRR8548615	UK - Background	Not recorded
SRR8650207	UK - Background	Not recorded
SRR8655352	UK - Background	Not recorded
SRR8657029	UK - Background	Not recorded
SRR8658167	UK - Background	Not recorded
SRR8705964	UK - Background	Not recorded
SRR8706124	UK - Background	Not recorded
SRR9260145	UK - Background	Not recorded
SRR9273867	UK - Background	Not recorded
SRR9422939	UK - Background	Not recorded
SRR9661630	UK - Background	Not recorded
SRR1963334	UK 2014-16	Unknown Destination
SRR1969268	UK 2014-16	Thailand
SRR1965676	UK 2014-22	Thailand
SRR3049829	UK 2014-22	Thailand
SRR3049215	UK 2014-30	Not recorded
SRR1968013	UK 2014-35	Middle East (region)
SRR1959406	UK 2014-51	Thailand
SRR7286930	UK 2015-20	Thailand
SRR7456855	UK 2015-22	Unknown Destination
SRR8730626	UK 2015-25	Thailand
SRR8730624	UK 2015-25	Asian continent
SRR7480298	UK 2015-28	Thailand
SRR7358345	UK 2015-31	Not recorded
SRR8711772	UK 2015-37	Vietnam
SRR3321865	UK 2015-38	China
SRR3322612	UK 2015-40	China
SRR7426916	UK 2015-49	Thailand
SRR7523079	UK 2016-01	Thailand
SRR3321867	UK 2016-02	Not recorded
SRR8703576	UK 2016-03	Vietnam
SRR7286957	UK 2016-04	Vietnam
SRR7465101	UK 2016-05	Not recorded
SRR7140618	UK 2016-11	Thailand
SRR8698412	UK 2016-13	Not recorded
SRR7458760	UK 2016-19	Not recorded

SRR7215983	UK 2016-29	Not recorded
SRR5193777	UK 2016-31	Asian continent
SRR7251050	UK 2016-43	Thailand
SRR7284509	UK 2016-45	Thailand
SRR8654764	UK 2016-45	Thailand
SRR7456852	UK 2016-45	Not recorded
SRR7249932	UK 2016-50	Not recorded
SRR5631907	UK 2017-02	Asian continent
SRR5632078	UK 2017-03	Asian continent
SRR5632883	UK 2017-05	Vietnam
SRR5631911	UK 2017-05	Thailand
SRR5583813	UK 2017-08	Thailand
SRR5632253	UK 2017-12	Thailand
SRR8647653	UK 2017-19	Not recorded
SRR7180042	UK 2017-29	Not recorded
SRR7417280	UK 2017-33	Not recorded
SRR8525982	UK 2017-42	Thailand
SRR8514519	UK 2018-08	Not recorded
SRR8509448	UK 2018-09	Not recorded
SRR8509391	UK 2018-10	Not recorded
SRR8508672	UK 2018-15	Not recorded
SRR7444196	UK 2018-24	Not recorded
SRR8401402	UK 2018-28	Thailand
SRR7850543	UK 2018-31	Thailand
SRR7841556	UK 2018-35	Not recorded
SRR8131564	UK 2018-41	Not recorded
SRR8293593	UK 2018-46	Not recorded
SRR8568731	UK 2019-06	Thailand
SRR8637744	UK 2019-06	Thailand
SRR8648307	UK 2019-06	Thailand
SRR8916308	UK 2019-14	Cambodia
SRR9287477	UK 2019-17	Not recorded
SRR9274332	UK 2019-18	Not recorded
SRR9274348	UK 2019-22	Asian continent
SRR10096412	UK 2019-35	Asian continent
SRR10096398	UK 2019-35	Not recorded
SRR10404552	UK 2019-43	Thailand
SRR10580024	UK 2019-47	Not recorded
SRR11048375	UK 2020-05	Asian continent
SRR11435813	UK 2020-12	Not recorded

SRR11550435	UK 2020-14	Vietnam
SRR11575805	UK 2020-14	Asian continent
SRR12626261	UK 2020-35	Not recorded
SRR10483510	SRR10483510_1	Not recorded
SRR10483546	SRR10483546_1	Not recorded
SRR10483454	SRR10483454_1	Not recorded
SRR10484327	SRR10484327_1	Not recorded
